# The interrater reliability of the Swedish version of the Structured Clinical Interview for the DSM-5 Alternative Model for Personality Disorders, Module I and Module II for diagnosing personality disorders among adults referred to specialized psychiatric care – a pilot study

**DOI:** 10.1186/s12888-025-07615-4

**Published:** 2025-11-14

**Authors:** Sophie Steijer, Steinn Steingrimsson, Niklas Meyerson, Peter Sand

**Affiliations:** 1 Department of Psychiatry, Sahlgrenska University Hospital, Gothenburg, Sweden; 2 Sahlgrenska Academy, Institute of Neuroscience and Physiology, University of Gothenburg, Gothenburg, Sweden; 3 Department of Psychology, University of Gothenburg, Gothenburg, Sweden

**Keywords:** SCID-5-AMPD, Personality disorder, LPFS, Psychometrics, Inter-rater reliability

## Abstract

**Background:**

There is an ongoing debate on which model for diagnosing personality disorders is optimal, with the DSM-5 Alternative Model for Personality Disorders being one of the most prominent. The Structured Clinical Interview for the DSM-5 Alternative Model for Personality Disorders (SCID-5-AMPD) is a semistructured diagnostic interview to guide the assessment of the three parts of the Alternative DSM-5 Model for Personality Disorders (hereafter referred to as the Alternative Model), as published in DSM-5 Section III. The structured interview has been tested and translated to several languages, however, not to Swedish.

**Objectives:**

To examine the psychometric properties of the Swedish translation of the Structured Clinical Interview for DSM-5 Alternative Model for Personality Disorders, Module I and Module II for treatment seeking persons with suspected personality pathology including reliability in a Swedish clinical context.

**Methods:**

This study of 38 outpatients with suspected personality disorder studied the test-retest interrater reliability (Intraclass correlation coefficient (ICC)), estimates of Cronbach’s alpha values in a clinical setting.

**Results:**

The main results were that ICC ranged between 0.1 and 0.56 for the domain scales of Modules I and II, whereas the estimates of scale reliability (α) ranged from 0.25 to 0.85.

**Conclusion:**

Despite the challenges posed by the limited number of raters and the small sample size, this study provides valuable insights into the application of the SCID-5-AMPD model within a Swedish context. While the results suggest that further exploration is needed to fully establish the model’s effectiveness and benefits in clinical practice, they also highlight the importance of proper training, clinical experience and calibration for achieving optimal reliability. Continued research is essential to refine the tool and assess its broader applicability. With appropriate enhancements in training and methodological rigor, the SCID-5-AMPD has the potential to significantly contribute to the assessment of personality disorders.

## Introduction

Personality Disorders (PDs) are common and associated with poor quality of life, high rates of healthcare consumption and low occupational participation [1, 2]. Therefore, the diagnosis of personality disorders is of great importance for clinical reasons, research and society in general [3–9].

The Alternative DSM-5 Model for Personality Disorders, published in DSM-5 Section III, is a hybrid model of personality disorder classification comprising both dimensional elements and categorical elements grounded in an argument for a new approach [10–14]. The dimensional components include ratings of the severity of impairment in personality functioning and ratings of the descriptiveness of 25 pathological personality trait facets organized into five trait domains. The categorical components include ratings of the potential presence of six specific personality disorders. The SCID-5-AMPD is a semi-structured diagnostic interview designed to guide the assessment of the three parts of the Alternative DSM-5 Model for Personality Disorders. The SCID-5-AMPD consists of three modules. Module I is used for the assessment of the Level of Personality Functioning Scale; Module II focuses on the assessment of the five trait domains and 25 pathological personality trait facets that are listed and defined in DSM-5. And Module III facilitates the evaluation of the categorical components of the Alternative Model, assessing Criterion A (required impairments in personality functioning) and Criterion B (required pathological personality trait facets) for each of the six specific personality disorders of the Alternative Model.

Since the publication of the DSM-5, there have been several studies focusing on the psychometric properties of the AMPD, including test-retest and inter-rater reliability (IRR) studies of the SCID-5-AMPD, Module I has been evaluated in Norwegian samples and both Module I and II have been studied in Italian samples [15, 16].

In addition to differences in language, location, and patient population, these studies differ in the raters’ characteristics and experimental setup.

While ascertaining the adequacy of the translation is the overall purpose, patient and rater factors affect the IRR. The prevalence of personality disorders may vary across patient populations - geographically and temporally - which directly affects estimands that include the variation in the population in their definition. This includes the intra-class correlation coefficient (ICC), which is commonly used as a metric of IRR. Similarly, raters’ characteristics, including overall clinical experience and level of training in SCID-5-AMPD, may influence the level of agreement, which directly affects the estimands.

Somma et al. [16] included an unspecified number of licensed clinical psychologists with 1–3 years of experience in personality disorder assessment as raters to minimize the influence of clinician experience on psychometric property estimates for the SCID-5-AMPD Modules I and II measures. The raters participated in courses organized by the Italian publisher of the DSM-5 and related tools, followed by six months of training for each interview instrument. During all interviews, both the interviewer and observer were present together and independently scored each patient.

Both experienced clinicians, inexperienced clinicians, and undergraduate students participated as raters in Christensen et al., with the experienced raters receiving a two-day workshop and subsequently training the other participating raters. Christensen et al. used a test-retest IRR design wherein 33 patients were interviewed twice, with a maximum of two weeks between interviews. The rater conducting the second interview was blind with respect to the first interview and rating. In addition to this test-retest IRR design, the assessments were video- recorded and a sample of 17 of these recordings was scored independently by multiple raters. This aspect of the study is similar to the design of Somma et al. wherein multiple raters observ the interview live but each patient is interviewed once. While scoring a video-recorded interview is a diffferent experience from live observation and therefore may affect reliability estimates, we refer to all single-interview with multipl-rater designs as single-test IRR.

Generally, single-test IRR and test-retest IRR do not target the same estimand. In other words, the design used to assess diagnostic reliability is crucial. Test-retest reliability involves two independent clinicians conducting separate interviews at different times, typically with an interval of one to two weeks. Thus, it includes the variability due to differences in clinicians conducting the interview and patients responding to these differences, in addition to the variability in rating an interview. As such, the test-retest method offers a more accurate estimate of diagnostic reliability and better reflects clinical practice, providing a more valid measure of diagnostic stability [19].

Chmielewski et al. [17] discussed the use of audio/video-recorded single-test IRR in comparison to the test-retest IRR method and identified several limitations of the audio/video-based IRR study design. These include the potential for artificially increased agreement [compared with the estimand of test-retest IRR], limited opportunities for the second rater to ask follow-up questions or clarify responses, possible missing information for the second rater, and the fact that the audio/video method may not accurately represent clinical practice, where clinicians might receive different patient narratives. Chmielewski et al. showed that test-retest designs produce lower reliability estimates than video/audio methods, but they better reflect clinical reality.

A disadvantage of the test-retest design is that the interviewee’s condition can change between the first and second interview. Especially in a clinical population, this is a concern, however choosing an appropriately short time between intervals should result in minimal clinically irrelevant changes. Furthermore, the test-retest design requires that patients agree to be interviewed twice, which may impact recruitment and costs. It also requires that the interviewee participates genuinely in both assessments and that there is no influence from the first assessment on the answers in the second assessment. During the second interview, a patient may recall the first interview and attempt to align their responses, or deliberately respond differently.

In addition to patient factors, raters may change over time (becoming more lenient or strict), or become fatigued, which can bias the results. Finally, the test-retest IRR studies often use small samples due to resource constraints, which reduces statistical power and generalizability.

The advantage of the test-retest design is that it allows for a direct estimation of the IRR as it would occur under clinical conditions [19, 21]. Specifically, it evaluates the consistency of diagnostic assessments when separate clinicians independently conduct the SCID-5-AMPD-I and II interviews at different times, offering a more valid measure of reliability than approaches relying on shared data sources such as video/audio recordings.

However, the strengths of the test-retest design must be understood within the practical context in which the SCID-5-AMPD interviews are administered. Although a necessary condition for achieving high reliability is that two raters interpret and apply patients’ responses consistently, this condition alone is not sufficient to arrive at the same assessment. In clinical settings, interviewers not only interpret but also conduct the interviews, meaning they actively shape the process and content of the assessment through their interactions with the patient. Clinicians may, based on their evolving understanding during the session, choose to ask more questions or probe deeper into particular areas. This variability introduces a significant potential for the interviewer to influence the interview content, which can, in turn, affect the outcomes.

Moreover, when a rater views a previously conducted interview (as in video- or audio-based IRR designs), the selection and phrasing of follow-up questions by the original interviewer can inadvertently communicate their interpretative stance or diagnostic judgment. These embedded cues may bias the second rater, reducing the independence of their assessment [28]. In contrast, the test–retest method better preserves rater independence, albeit with its own sources of variability.

Additionally, patient responses are not always stable across interviews. Patients may offer different information to different interviewers, even when the same questions are asked, due to differences in interpersonal dynamics, perceived empathy, or subtle non-verbal cues. Thus, variability in patient disclosures further complicates the reliability assessment, though it more accurately reflects the complexities encountered in everyday diagnostic practice.

In summary, while the test–retest method introduces potential variability due to interviewer influence and patient inconsistency, it offers a more clinically relevant estimation of diagnostic reliability in the context of the SCID-5-AMPD. As such, it is considered particularly suitable for assessing the reliability of semi-structured interviews used in dimensional models of personality pathology, such as the Alternative Model for Personality Disorders (AMPD).

We aimed to evaluate the inter-rater and test-retest reliability (IRR) with 95% confidence intervals. If the SCID-5-AMPD-I and II achieve high IRR, this would demonstrate both temporal stability and inter-rater agreement, thereby supporting that the SCID-5-AMPD measures consistent dimensions of personality pathology. However, the study design does not clarify whether low IRR is due to poor temporal stability, poor inter-rater agreement, or both.

An important aspect is to test the SCID-5-AMPD in different languages and contexts to ensure the global applicability of the SCID-5-AMPD.

Regarding our study, it is reasonable to apply the criteria used for the Intraclass Correlation Coefficient (ICC) estimates in the DSM-5 field trials, based on a test-retest design with two raters [17–19]. In summary, the DSM-5 alternative model for personality disorders has shown promise but needs to be tested further to ensure reliability and feasibility for clinicians and patients.

## Objectives

The overall research aim of this study is to examine the psychometric properties of the Swedish translation of the Structured Clinical Interview for DSM-5 Alternative Model for Personality Disorders Modules I II (SCID-5-AMPD) for treatment-seeking persons with suspected personality pathology. The focus is to test the reliability (i.e. clinical consistency) which captures the extent to which clinicians arrive at the same diagnostic conclusion, when using the SCID-5-AMPD and having access to the same diagnostic information in a clinical setting.

## Materials and methods

### Clinical sample

The region of Västra Götaland and the city of Gothenburg are on the west coast of Sweden with a population of 1,713,907 as registered in 2020. The region contains both larger urban and rural areas. From January 2020 to June 2021, 40 individuals were included in this pilot study, with two who withdrew consent and therefore 38 participants completed participation. All participants in the test-retest study were recruited from the Gothenburg area at an outpatient clinic specialized in the care of personality disorders at Sahlgrenska University Hospital.

At the participating psychiatric clinic, the current gender distribution for patients with personality disorders is 78% female and 22% male, similar to this study (79% female and 21% male).

### Setting

Inclusion criteria for the participants were: ongoing follow-up at the Department of Psychiatry at Sahlgrenska University Hospital, Gothenburg, and fluency in Swedish. Ongoing follow-up was defined as being actively engaged in care after referral, either by a healthcare professional or self-referral, for a suspected personality disorder. All referrals were initially examined by a referral team to ensure that the appropriate specialist team was involved. Participants with Schizophrenia-spectrum disorder, sequelae after brain injury, pervasive developmental disorders such as autism spectrum disorder or mental retardation and ongoing substance use disorders or unhealthy alcohol use were excluded. All measures except the SCID-5-AMPD were administered as part of the routine clinical assessment.

### Raters and training

Participants underwent structured interviews as part of their clinical evaluation with the SCID-5-AMPD Module I and Module II interviews [22–24], performed by two raters: one was a psychologist with one year of clinical experience who had received four hours of informal training in the interview, including watching a video presentation from an established interviewer. The other rater was an experienced psychiatrist who had completed a four-day workshop training on Module I. The SCID-5-AMPD training was overseen by clinicians with APA-approved training and authorization. The psychiatrist had used the SCID-5-AMPD in clinical practice for one year prior to the study.

Formal training in the use of SCID-5-AMPD, Module II was not provided, but its content was reviewed during consensus meetings. Both raters relied on clinical experience and knowledge from the patient´s psychiatric history and medical records. The SCID-5-AMPD raters alternated between the first and the second interview positions, and were kept blind to each other’s results.

One of the researchers (PS) informed the patients about the study via phone and provided written information during a scheduled meeting. At this meeting, the patients received a list of pre-booked appointments with raters and a psychologist for a SCID-II interview. Each interview was scheduled at one-week intervals.

### Participants

In total, 38 participants were assessed separately by two raters with the Swedish translation of the semi-structured interviews, the SCID-5-AMPD Module I, Module II and by one of three psychologists with the SCID-II. The SCID-5-AMPD Module I and Module II were administrated separately by two raters and were scheduled at one-week intervals. In practice, the actual intervals varied: most participants were re-assessed after 7 days (*n* = 17), while others had shorter (2–6 days) or longer intervals (8–35 days). In two cases, the interval was substantially longer (55 and 122 days). The mean interval between the two ratings was 13.1 days (SD = 20.8; range: 2–122 days; *N* = 38). This interval was chosen to minimize recall effects while avoiding the risk of substantial clinical change, in line with recommendations for test–retest reliability studies (Kraemer et al., 2012).

### Outcome measurements

#### Diagnostic assessment DSM-5 section II personality disorders

During the study, all patients were assessed with the Structured Clinical Interview for Axis II Disorders (SCID-II) which is the current standard for assessing PDs (Table 1). The SCID-II is a semi-structured interview used to assess the 10 DSM-5 Section II personality disorders and personality disorder NOS [20] which has shown good interrater and test-retest reliability in PD samples [21]. Three clinical psychologists were trained in performing the SCID-II through courses arranged by the Psychology department at the Gothenburg University and the department of psychiatry at the Sahlgrenska University Hospital. A PD NOS diagnosis was made if the participants were rated with ten or more SCID-II criteria but without meeting the criteria for any specific PD diagnosis. The results of the SCID-II ratings were kept blind to the raters conducting the SCID-5-AMPD evaluations, and none of the SCID-5-AMPD raters took part in the SCID-II assessments.

#### Diagnostic assessment for alternative model of personality disorders, DSM-5, section III personality disorders

Prior to the study, the APA Publishing granted permission to reproduce items from the original version of the SCID-5-AMPD Module I and Module II for use in our study only (PL16353). Use was restricted to English and Swedish. The original version of the SCID-5-AMPD, Module I and Module II [22, 23] was translated into Swedish using forward and backward translation by the authors (SoSt, PS and a bilingual translator). The Swedish version of the modules was then reviewed through structured patient interviews to identify any translation difficulties. Some minor changes were made and implemented (NM, PS, SoSt). During the study, all patients were assessed using the Structured Clinical Interview for DSM-5 Alternative Model Personality Disorders Module I and Module II (SCID-5-AMPD) [22, 23].

#### The SCID-5-AMPD, Module I

The Structured Clinical Interview for DSM-5 Alternative Model Personality Disorders, Module I (SCID-5-AMPD), is a semi-structured instrument for assessing the four domains of personality functioning presented in the Level of Personality Functioning Scale: identity, self-direction, empathy and intimacy. These domains are typically impaired in personality disorders [22]. Each domain is devided into three subdomains, which are rated on a five-point scale: level 0= “little or no impairment,” level 1= “some impairment,” level 2= “moderate impairment”, level 3=” severe impairment” and level 4 =” extreme impairment”.

Personality functioning and adaptation are characterized by how individuals think about and understand themselves and their interactions with others.

The SCID-5-AMPD, Module I begins with a general overview covering demographic data, education, work history, and current and past periods of psychopathology. Prior to the interview, raters were advised to read the patient’s psychiatric history from their medical record. The Module I continues with eight preliminary questions to get a basic sense of the interviewee’s view of self, basic approach to life and quality of interpersonal relationships.

After having considered the patients’ responses to the eight preliminary questions, the estimated global LPFS score, along with the patients’ response to the subdomain’s screener questions, the clinician begins the interview at the level where they believe the individual may be functioning.

The interview continues with an assessment of the twelve subdomains of personality functioning presented in the LPFS. For each subdomain, the clinician asks screener questions and seeks examples, elaborating until sufficient information is gathered to make a rating. The clinician should proceed to ask questions corresponding to increasingly severe levels of impairment until the patient no longer qualifies for that level.

The goal is to determine the level of functioning for that subdomain. Each level of functioning is defined according to the LPFS. The manifestations observed from the interview should reflect the patient’s overall personality functioning. Impairment in functioning may vary based on the individual’s emotional state; for instance, a person’s ability to empathize may decrease significantly when they are upset. Consequently, the overall rating for that subdomain should consider how often the person experiences emotional distress. All information collected throughout the assessment of the twelve subdomains, along with the interaction with the individual, contributes to determining the level of personality functioning. The clinician records each subdomain and domain score, totals the scores, and then averages them to obtain the overall level of personality functioning, ranging from 0 to 4.

#### The SCID-5-AMPD, Module II

The Structured Clinical Interview for DSM-5 Alternative Model Personality Disorders Module II is a semi-structured instrument for rating the descriptiveness of twenty-five pathological personality trait facets grouped into five trait domains: Negative Affectivity, Detachment, Antagonism, Disinhibition and Psychoticism. A personality trait is a tendency to feel, perceive, behave, and think in relatively consistent ways across time and across situations in which the trait may be manifested. All individuals can be located on the spectrum of trait dimensions. The personality traits model [23, 25] comprises 25 specific personality traits, also known as facets, that are maladaptive variants of the five domains of the validated and replicated personality model known as “FiveFactor Model of Personality.” The 25 specific personality traits represent a list of personality facets chosen for their clinical relevance. Although traits do change throughout the life span, they show relatively consistency compared with symptoms. A person may behave impulsively at a specific time for a specific reason, but it is when behaviors aggregate across time and circumstance such that a pattern of behaviors distinguishes between individuals, that they reflect traits. Even people who are impulsive are not acting impulsively all the time. Each personality trait is described with questions and the rater will ask all the questions for each personality trait and make a single rating of the degree to which each trait describes the interviewee. Each personality trait within each domain is rated on a four-point scale of descriptiveness from 0 = “very little or not at all descriptive” to 3 = “very descriptive.” The personality traits should describe the patient’s current personality and be descriptive for at least the past 2 years. At the end of each domain the rater records the scores in that domain and calculates a total score and an average score for the domain. The total summed score for each domain is used to provide a profile of the interviewee’s personality in terms of domains and a summary of average scores.

In summary, all the information gathered prior to and throughout the interview and the interaction with the patient serve as data in determining the level of personality functioning and the configuration of the characteristic pathological personality traits [22–24]. This data facilitates to assessment of the six specific diagnoses personality disorders that are included in the AMPD.

### Ethical approval

All patients participated voluntarily in the study after being presented with a detailed description and signing a written informed consent form to take part in the study. The study protocol was reviewed and approved by the Swedish Ethical Review Authority (DNR: 2019–04189).

### Design and statistical analysis

Descriptive statistics were calculated for the demographic variables sex, age, education, marital status, occupation, and diagnosis. Age is shown as mean and range. All other categorical variables are presented as numbers and percentages.

Two statistical methods were used to evaluate the SCID-5-AMPD, Module I and Module II inter-rater reliability: intraclass correlation coefficient for dimensional scores and Cronbach’s alpha to estimate the scale reliability [26, 27]. The ICC for absolute rater agreement was used to assess the IRR of the SCID-5-AMPD, Module I and Module II scores for the total LPFS, domains, subdomains and for the pathological personality traits domains and subdomains. In the test-retest IRR study, the form and type of ICC was a single-measure, absolute-agreement, where a two-way mixed-effect model was applied. Each participant was rated by two raters. Raters were included as a fixed effect. Participants were not randomly selected, instead all eligible patients were included. Nonetheless, participants were included as a random effect. For the test-retest IRR study, we provide information about the model, form and type of ICC calculated according to McGraw and Wong [26]. The ranges are as follows: excellent (>0.75), good (0.60–0.75), fair (0.40–0.59) and poor (0.40). For the test-retest field trials, the DSM-5 Task Force regarded ICC values of >0.40 with a maximum width of 0.50 for the 95% confidence interval as acceptable [19].

Cronbach’s alpha was calculated for each rater and domain to provide an estimate of the scale reliability of Modules I and II [27].

The statistical analysis was conducted using SPSS Version 28.0 (IBM Corp. Released 2021. IBM SPSS Statistics for Windows, Armonk, NY: IBM Corp).

## Results

### Demographics

In total, 38 participants were included, of whom 73% received a diagnosis of personality disorder and specifically 42% borderline personality disorder according to the WHO ICD-10 classification (Table 1). Among the participants 58% were on sick leave or were part-time students and 45% lived in a single household. The majority were female (79%). The mean age was 28 years, and most had finished more than primary and high school education (76%). (In 2022, 79.7% of students in national programs in Sweden completed high school with a diploma within three years.) All 38 patients (100%) were diagnosed with at least one comorbid mental disorder in ICD-10. The most frequent diagnosis mixed anxiety and depressive disorder (40.6%), followed by dysthymia (23.0%), recurrent depressive disorder (7.8%), moderate depressive disorder (5.2%), persistent mood disorder (5.2%) followed by obssessive-compulsive disorder (2.6%), panic disorder (2.6%), post-traumatic stress disorder (2.6%), social phobia (2.6%), generalized anxiety disorder (2.6%) and disturbance of activity and attention (5.2%).

**Table 1:** Demographics of study participants

Variable	Total (*n* = 38)
Sex	
Female	30 (79%)
Male	8 (21%)
Mean age (range)	28.4 years [19–43]
Education	
Primary school	9 (24%)
High school	20 (52%)
University degree	9 (24%)
Marital status	
Single household	17 (45%)
Cohabiting with partner	10 (26%)
Cohabiting with another person	11 (29%)
Occupation	
Student	9 (24%)
Professional	16 (42%)
On sick leave	13 (34%)
Personality disorder according to SCID-II	
Borderline personality disorder	16 (42%)
Personality disorder NOS	6 (16%)
Avoidant personality disorder	3 (8.2%)
Histrionic personality disorder	1 (2.6%)
Schizoid personality disorder	1 (2.6%)
Dependent personality disorder	1 (2.6%)
Other mental disorder	10 (26%)

### Inter-rater reliability

One patient was identified as an outlier based on a large discrepancy between raters across multiple domains and was excluded from the final ICC analysis; this exclusion reduced the width of some confidence intervals, particularly in Module I. For Module I, the ICC values on the domain scales ranged from poor ICC 0.25–0.34 (Empathy and Intimacy domains) to fair 0.49–0.56 (Identity and Self-direction domains). The ICC 0.57 was obtained for the total scale LPFS which is fair (Table 2). On the subdomain level, poor ICCs were detected for all Empathy subdomains (0.10–0.38), such as for “tolerance of differing perspectives” (-0.10) and for two Intimacy subdomains “depth and duration of connections with others” (0.07) and for “desire and capacity for closeness” (-0.07). Most other subdomains ICCs were fair (0.43–0.53). The breadth of the 95% CIs varied considerably, ranging from relatively narrow intervals (e.g., LPFS total: 0.30–0.75) to wide intervals spanning negative to positive values (e.g., “tolerance of differing perspectives”: −0.10–0.38). Wider CIs were generally associated with lower ICC values and greater rater variability. In this study, the term “range” refers to the variability of ICC values across domains, not to the range of their confidence intervals.

Because the test–retest interval varied across participants (range: 2–122 days), we examined whether this variation influenced reliability estimates by calculating the correlation between the test–retest difference scores on the LPFS and the interval length. The association was small and non-significant (*r* = .20, 95% CI [–0.13, 0.49], *N* = 38), suggesting that interval length did not substantially affect the results.

**Table 2:** ICC and CI: s - SCID-5-AMPD - Module 1

	Rater 1			Rater 2				
SCID-5-AMPD Module 1 Scales	M	SD	α	M	SD	α	ICC	CI
Domain Scale Scores								
Identity	2.59	0.55	0.75	2.72	0.60	0.71	0.49	0.21/0.70
Self-direction	2.46	0.55	0.72	2.51	0.64	0.70	0.56	0.30/0.75
Empathy	2.67	0.50	0.81	2.59	0.68	0.75	0.25	/-0.08/0.52
Intimacy	2.81	0.45	0.69	2.84	0.75	0.85	0.34	0.02/0.59
Levels of Personality Functioning Scale	2.63	0.45	0.91	2.66	0.54	0.88	0.57	0.30/0.75

For Module II, the ICC on the domain scales ranged from poor 0.10–0.29 (Psychoticism and Antagonism) to fair 0.49–0.56 (Disinhibition, Detachment, Negative Affectivity) (Table 3). On the subdomains level, poor ICCs were detected for all antagonism subdomains (-0.07- 0.42), and there is rater disagreement for the subdomains “unusual beliefs and experiences”, eccentricity”, “cognitive and perceptual dysregulation” (ICC range: 0.07–0.14). Confidence intervals for Module II showed similar patterns to those in Module I, with narrower CIs for domains with higher ICCs and wider CIs for domains with poor rater agreement.

**Table 3:** ICC and CI: s - SCID-5-AMPD - Module 2

	Rater 1			Rater 2				
SCID-5-AMPD Module 2 Scores	M	SD	α	M	SD	α	ICC	CI
Domain Scale Scores								
Negative Affectivity	1.78	0.33	0.25	1.74	0.50	0.65	0.56	0.27/0.76
Detachment	1.73	0.45	0.63	1.34	0.69	0.81	0.51	0.13/0.74
Antagonism	1.41	0.50	0.73	0.70	0.69	0.85	0.29	/-0.09/0.60
Disinhibition	1.40	0.56	0.66	1.18	0.78	0.76	0.49	0.21/0.70
Psychoticism	0.20	0.32	0.38	0.41	0.45	0.54	0.10	/-0.17/0.37

Mean (M) and standard deviation (SD) of domain scale scores per rater together with Cronbach’s alpha (α) for each rater and domain, were calculated to estimate the scale reliability, and intraclass correlation coefficient (ICC) as a measure for absolute rater agreement. The ICC ranges are as follows: excellent (> 0.75), good (0.60–0.75), fair (0.40–0.59) and poor below (0.40).

### Scale reliability

For Module I, the domain scale scores exhibited acceptable reliability estimates of Cronbach’s alpha values, ranging from 0.69 to 0.85, for both raters. The total scale score for the Level of Personality Functioning Scale, Module I, demonstrated robust reliability with Cronbach’s alpha values of 0.88 and 0.91 (Table 2). In Module II, the domain scale scores generally exhibited satisfactory reliability estimates of Cronbach’s alpha values (ranging from 0.63 to 0.85), though exceptions were noted for the Psychoticism and Negative Affectivity scales, where lower estimates of 0.25–0.54 were observed. Notably, in Module II, variations between raters were observed, particularly in the Negative Affectivity and Psychoticism trait domains, where Cronbach’s alpha for Rater 1 was lower than that for Rater 2 (Table 3). It’s important to acknowledge that these reliability assessments were based on a relatively small sample size, and caution is advised when interpreting the results, especially in domains with lower estimates.

## Discussion

This study examined the psychometric properties of the Swedish version of the LPFS assessed by the Structured Clinical Interview for the DSM-5 Alternative Model for Personality Disorders (the SCID-5-AMPD), Module I and pathological personality traits assessed by the SCID-5-AMPD, Module II [22–24]. This study is the first to evaluate the psychometric properties of the Swedish SCID-5-AMPD Module I and II, thereby enabling assessment for research and clinical purposes.

The test-retest intraclass correlation coefficient (ICC) levels were poor to fair, and the ICCs ranged from 0.25 to 0.56 for the LPFS domains, Empathy, Intimacy, Self-direction and Identity. The ICC was 0.57 for the LPFS total score. The findings indicate a moderate level of agreement in the measurements of the LPFS domains, revealing notable variability between the two raters as well as in how the measurements relate to the clinical setting. However, when we examine the results based on a 95% confidence interval, several of the domains (Empathy and Intimacy) fall below the ICC’s requirements for acceptable reliability (ICC > 0.4, CI upper limit < 0.5), and the remaining domains are precariously close to the threshold. This indicates a relatively poor agreement between the two assessors. Compared to the results from the Norwegian study, our results are considerably worse. This could possibly reflect cultural differences, issues with translation, and other problems with the assumptions and design of this study. Based on our experience, the Norwegian and Swedish clinical practice are closely aligned, and such an explanation would not be likely. We have followed the convention for translating an instrument from one language to another using forward and backward translation, and the translation has been reviewed by several different experienced clinicians. Therefore, we find it unlikely that the discrepancy in the results would be due to translation flaws.

Similarly, we do not expect major differences in the prevalence of personality disorders.

### The patient sample characteristics and relevance

Our study along with those by Buer Christiansen [15], Somma [16], and Ohse [32], each explored personality disorder assessment using the SCID-5-AMPD. However, they differed in patient sample characteristics, diagnostic distribution, and clinical contexts, which provide insight into the psychometric application of the AMPD framework.

Our study (*n* = 38) recruited participants from a specialized public outpatient psychiatric clinic for personality disorders. The sample was predominantly female (79%) and relatively young **(**M = 28 years). Clinically, the sample was highly impaired, all patients were treated with psychotropic drugs and 73% met criteria for at least one DSM-5 personality disorder, most commonly borderline personality disorder (42%), followed by personality disorder NOS (16%) and avoidant personality disorder (8.2%). Additionally, 100% of the sample received a co-occurring symptom disorder diagnosis, with mixed anxiety and depressive disorder (40.6%) and dysthymia (23%) being the most frequent. These findings provide further justification for employing the SCID-5-AMPD in the assessment of diagnostically complex and high-risk populations within clinical environments.

Buer Christensen et al. (*n* = 33) included a sample demographically similar to ours,73% women, M = 29 years with moderate education levels. Diagnostically, borderline personality disorder (46%), personality disorder NOS (29%), and avoidant personality disorder (21%) were most prevalent. Notably, 85% of the sample had comorbid Axis I disorders, including recurrent depression (42%), social phobia (24%), and PTSD/dysthymia (12%). The sample’s high comorbidity and clinical complexity underscore its appropriateness for evaluating test-retest inter-rater reliability of the SCID-5-AMPD tools.

Somma et al. (*n* = 88) employed a less clinically impaired sample, comprised of psychotherapy-seeking individuals who self-identified with emotional and interpersonal difficulties. The sample was more demographically balanced **(**54.5% women, M = 36.47 years) and relatively well-functioning—78.5% had at least a high school diploma, and 62.5% were married. The distribution of personality disorders was broader but less severe: borderline personality disorder (19%), narcissistic personality disorder (18.2%), obsessive-compulsive personality disorder (13.6%), and avoidant personality disorder (13.6%). Comorbidity data were not detailed, but the population appears comparatively lower in symptom severity. The study offers insights into SCID-5-AMPD performance in general outpatient settings.

Ohse et al. (*n* = 121) conducted their study as part of the Berlin Study of the AMPD, recruiting 79.3% of participants from outpatient psychotherapy services and 17.4% from psychiatric inpatient units. The sample was predominantly female (71.1%), employed (75.2%), and relatively young **(**M = 32.2 years). Clinically, the sample showed high psychiatric comorbidit**y**, with 54.6% screening positive for depressive symptoms, 50.0% for anxiety, and 33.6% on psychotropic medication. According to the SCID-5-AMPD, 46.6% met criteria for at least one personality disorder, most frequently borderline (16.4%), obsessive-compulsive (15.5%), and avoidant (14.7%). Regarding personality functioning (SCID-5-AMPD-I), 43.0% met the 1.5 threshold and 33.1% the stricter 2.0 threshold for personality disorder. The distribution of impairment ranged from no impairment (10.7%) to extreme impairment (2.5%). This diagnostically heterogeneous sample is especially useful for evaluating criterion A of the AMPD.

Together, these four studies reflect the adaptability of the SCID-5-AMPD instruments within a range of clinical environments. Our study and Buer Christensen focused on clinically complex samples, facilitating comprehensive evaluations of diagnostic reliability. Somma’s research illustrated the utility of the SCID among moderately impaired outpatient psychotherapy clients, while Ohse’s study integrated both inpatient and outpatient populations, spanning the continuum of care. Notably, borderline personality disorder was consistently identified as the most prevalent condition across all studies, and the observed variability in psychiatric comorbidity emphasizes the necessity of considering functional impairment when interpreting SCID-5-AMPD results.

That highlights another aspect of this specific study’s design, namely that the same two assessors interviewed all participants. The study estimates the IRR between these two assessors, which limits the ability to generalize results to other pairs of raters. Variability in IRR may occur with different pairs of assessors, and there is no indication of how this pair compares to others in Sweden. Differences in IRR might arise between practitioners from different clinics or regions, or due to varying levels of clinical experience. While other studies emphasize the need for training, clinical use of instruments is not always preceded by rigorous training criteria.

Upon examining Table 2, it becomes evident that the patient sample is characterized by severe impairment in personality functioning. Both assessors reported mean LPFS scores around 2.6–2.7 with small standard deviations, indicating a relatively homogeneous sample in terms of impairment level. As Kraemer and Kupfer (2006) noted, it is challenging to obtain high correlation coefficients when the standard deviations are small.

The contribution of this study is that this presumed reliable instrument, in individual cases, still allows room for the individual clinician’s judgment in interpreting the responses.

Evaluating an individual for a suspected Personality Disorder (PD) poses distinct challenges. The clinician must employ various methods to gather information for a clinical diagnosis, such as direct questioning, observation, and interaction with the patient. However, these comments prompts consideration about the potential limitations of relying heavily on interview scores in clinical practice, emphasizing the importance of other data sources and expressing concerns about the time investment required for ratings (which unfortunately are not provided).

### Problems in clinical interviewing

Evaluating a patient for a personality disorder is complicated by the inherent subjectivity of self-perception, with individuals providing different responses at different times [28]. The SCID-5-AMPD interview is posited to afford clinicians the opportunity to discern patterns in the patient’s self-description, romantic relationships/friendships, and school/work functioning. These domains aim to paint a comprehensive picture of personality functioning and traits. Additionally, clinicians must rely on their observations of patient interactions during evaluation interviews [29]. However, a critical perspective questions whether the SCID-5-AMPD interview offers unique advantages in discerning patterns compared to alternative methods, emphasizing the need for evidence supporting its distinctiveness.

### Direct questioning

In our study, we noted that patients tended to reply “sometimes” when asked whether they “often” had a particular experience question which made the evaluation somewhat difficult. A semi-structured interview like SCID-5-AMPD is useful clinically for communication with others, description of patient’s problems and treatment planning [30]. For that purpose, it is important to ask patients to describe in detail their relationships and functioning across several different areas of life over the past ten years in different situations with several people [24].

According to the User’s Guide for the SCID-5-AMPD [31] has “the clinician’s assessment should consider both the patient’s past and present history, and include follow-up questions intended to encourage the patient to expand on their answers. For each question the clinician should ask for examples and elaborate until they have sufficient information to make a rating. It is important to keep in mind that many individuals lack extensive, if any, insight regarding their personality functioning.” Clinicians must rely on their own judgment and clinical experience.

For example, the personality trait domain Psychoticism showed a low level of agreement (ICC = 0.10) when the same interview questions were asked of the same patient when two raters were involved? The assessment may be influenced by several factors, e.g., differences between clinicians, and the fact that the person, even if he or she is asked the same questions, can tell different things to the two interviewers, resulting in different assessments or measurement errors.

The ICC results in this study are lower than those compared with other studies [15, 16, 32]. An explanation could be the low number of raters (*n* = 2) and another explanation could be the choice of study design in a clinical setting. Partly unsatisfying results should not be taken as inherent qualities of the instrument but should be considered as influenced by other aspects in which this study has been conducted.

All domain scale scores reached or exceeded acceptable reliability estimates of Cronbach’s alpha values (0.69–0.85) for both raters with two exceptions for rater 1 for the Negative Affectivity and the Psychoticism trait domains. It is important to note that the reliability assessments were conducted with a relatively small sample size, which may impact the generalizability of the findings. Caution is advised when interpreting these results, especially considering the potential influence of sample size on reliability estimates. Results shows greater differences between raters, with Cronbach’s alpha for Rater 1 being lower than for Rater 2 in the trait domains Negative Affectivity and Psychoticism. In the present study, the reliability for the domain Empathy and the subdomain “tolerance of differing perspectives” estimates of ICC were respectively 0,25 and − 0.10. It assesses the individual capacity to consider and understand others’ alternative experiences without being too self-centered. To do so requires some self-criticism and a capacity to appreciate others’ opinions. Someone might endorse being good at understanding other perspectives but be unable to give supporting examples. However, the level of personality functioning, and the pathological personality traits were measured with a sufficient degree of IRR when assessed by the SCID-5-AMPD, Module I and Module II.

### Implications

Raters need training before the interview is implemented in practice. Overall, the Swedish version of the SCID-5-AMPD Module I and Module II had acceptable Cronbach’s alpha values. The inter-rater reliability is less than satisfactory. However, further studies are needed since other existing instruments have generally not been satisfactory in clinical practice.

## Strength and limitations

One of the primary strengths of this study is the use of a test-retest design, which is crucial for evaluating reliability over time. Another strength was the low dropout rate, which may reflect highly motivated patients. Furthermore, the group of participants was homogenous in terms of diagnoses since patients with severe psychiatric comorbidities were excluded. Another strength relates to the field of diagnostic assessment. Participants had previously undergone an initial psychiatric evaluation that indicated a suspected personality disorder, and were awaiting further investigation. Previous test-retest studies have employed either simultaneous observation by two interviewers or a video-recording design, both of which may introduce potential sources of bias. A blinded design was used to prevent bias, as raters were kept unaware of each other’s assessments throughout the study. However, this also meant that no formal case conferences or calibration meetings were held during the data collection period, which may have contributed to the comparatively lower ICC estimates observed (17).

There are also several noteworthy limitations in this pilot study. The most significant limitation is the use of only two raters and the small sample size (*n* = 38). Due to the limited sample size and the number of raters involved in the test-retest assessment, the generalizability of the results regarding the reliability of these instruments is questionable. The decision to include only two raters was driven by resource constraints and the exploratory nature of this pilot study. Another limitation is the missed opportunity to assess diagnostic reliability using video recordings, which could have ensured the correct use of the instrument. Comparing single-test IRR and test-retest IRR results provides some insight into the sources of variability, such as differences in interview performance (question choice, non-verbal cues) and patient-related factors or (e.g., varying levels of motivation) as well as differences in interpretation of answers.

The variability due to the interval between the two interview sessions is another limitation. The second interview did not always occur at the scheduled time, and an interval of over two months could potentially affect the results. Interviews conducted at different times might reflect the participant’s daily state, which could influence the outcomes. We did not control for interval length in the main analyses; however, an exploratory correlation analysis suggested that interval variation did not substantially affect the results. Additionally, differences in interview style between the two raters may have introduced further variation.

The sample included a high proportion of Borderline PD (42%), Avoidant PD (8.2%), and 2.6% each for Histrionic, Schizoid or Dependent PD with PD NOS (16%). The study did not include Narcissistic, Antisocial and Schizotypal Personality Disorders, which limits the generalizability of the findings to other personality disorders.

Furthermore, the study did not assess whether participants had previously undergone psychotherapy or counseling. Such interventions could potentially serve as protective factors, influencing the symptoms of suspected personality disorders. The absence of this information limits our understanding of how prior therapeutic experiences might affect the assessment outcomes. Future research should address these factors to provide a more comprehensive understanding of their impact on assessment outcomes.

## Recommendations

For the SCID-5-AMPD interview, we recommend training and clinical experience for all clinicians. Future studies should involve multiple assessors from different psychiatric clinics to enhance generalizability and precision, allowing subgroup analyses. Incorporating video recordings in test-retest designs is advised. If an experiment yields acceptable IRR estimates, rescoring may not be needed, but these recordings remain invaluable if results differ.

## Conclusions

This study represents the initial effort to translate and implement a valid measure of the Swedish SCID-5-AMPD, encompassing both Module I and Module II in a clinical setting. Despite the challenges related to the study design, such as the involvement of only two raters with limited training, the findings underscore the impact of training on the reliability of the SCID-5-AMPD. While the primary aim of the study was to assess interrater reliability, the results highlight the importance of comprehensive training. Future studies in the Swedish context are essential for a thorough exploration of the instrument’s psychometric properties, including both reliability and validity. Despite the less-than-ideal ICC reliability, the domain scale scores in Module I demonstrated a commendable level of reliability, surpassing acceptable reliability estimates of Cronbach’s alpha values (ranging from 0.69 to 0.85). Similarly, in Module II, the domain scale scores achieved satisfactory reliability estimates of Cronbach’s alpha values (ranging from 0.63 to 0.85). However, exceptions were noted for the Psychoticism and Negative Affectivity scales, where reliability estimates ranged from 0.25 to 0.54. It is important to highlight that the reliability assessments were conducted with two raters and a relatively small sample size, which may impact the generalizability of the findings.

In summary, while the SCID-5-AMPD shows potential, it is crucial to address the study’s limitations, particularly those related to study design. Proper training and calibration among clinicians are necessary to enhance the tool’s reliability and applicability in clinical practice.

## Data Availability

The datasets generated and/or analyzed during the current study are not publicly available due to privacy and confidentiality concerns. This decision was made to protect the sensitive information of the participants involved in the study and to adhere to ethical guidelines regarding data handling and protection. However, the data are available from the corresponding author upon reasonable request, ensuring that researchers can access the information for further analysis while maintaining the privacy of the individuals involved.

## Abbreviations

AMPD: Alternative Model for Personality Disorders
DSM-5: Diagnostic and Statistical Manual of Mental Disorders, 5th Edition
ICD-10: International Classification of Diseases, 10th Revision (WHO)
ICC: Intraclass Correlation Coefficient
IRR: Interrater Reliability
LPFS: Level of Personality Functioning Scale
PD: Personality Disorder
SCID-5-AMPD: Structured Clinical Interview for DSM-5 Alternative Model for Personality Disorders
SCID-II: Structured Clinical Interview for DSM-IV Axis II Personality Disorders
